# Anesthetic management using desflurane and nitrous oxide in a child with non-ketotic hyperglycinemia: a case report

**DOI:** 10.1186/s40981-024-00762-9

**Published:** 2024-12-27

**Authors:** Akifumi Mashima, Kenta Furutani, Hiroshi Baba

**Affiliations:** Department of Anesthesiology, Niigata University Medical and Dental Hospital, 1-754 Asahimachi-Dori, Chuo-Ku, Niigata, 951-8520 Japan

**Keywords:** Bispectral index, Desflurane, General anesthesia, Nitrous oxide, Non-ketotic hyperglycemia

## Abstract

**Background:**

Non-ketotic hyperglycinemia (NKH) is a rare autosomal recessive disorder caused by defects in the glycine cleavage system, leading to elevated glycine levels in the central nervous system. NKH manifests in various forms, with the neonatal type being the most severe and often associated with high mortality and significant neurological impairment. This case report highlights the successful uses of desflurane and nitrous oxide for anesthetic management in a patient with NKH.

**Case presentation:**

A 6-year-old girl with severe NKH, who had a history of delayed emergence from sevoflurane anesthesia, underwent tracheostomy for recurrent upper airway obstruction and severe obstructive sleep apnea. To address the previous issues with sevoflurane, general anesthesia was induced with propofol and fentanyl and maintained with 4% desflurane and 60% nitrous oxide. The electroencephalogram (EEG) showed near-complete suppression upon induction, which gradually resolved. Following cessation of desflurane and nitrous oxide, the patient exhibited early recovery, with eyes opening 3 min later and spontaneous breathing restored 19 min later. The patient experienced no postoperative complications and was discharged on the 14th postoperative day.

**Conclusion:**

This case suggests that desflurane, with its favorable pharmacological profile, may offer a superior alternative to sevoflurane for anesthetic management in NKH patients, particularly those with a history of delayed emergence. The observed EEG suppression may indicate heightened sensitivity to anesthetics in NKH, highlighting the need for tailored anesthetic strategies in this population.

## Background

Non-ketotic hyperglycinemia (NKH), also known as glycine encephalopathy, is a rare autosomal recessive disorder resulting from defects in the glycine cleavage system. This disorder leads to elevated levels of glycine in the central nervous system, manifesting in a variety of clinical forms [1]. The prevalence of NKH varies significantly by region, with higher incidences reported in northern Finland, British Columbia, and Israel (approximately 1 in 12,000 to 1 in 63,000 live births) [1, 2], compared with much lower rates in Japan (approximately 1 in 500,000 to 1 million births).

NKH presents with symptoms including seizures, hypotonia, hiccups, and developmental delays. The condition manifests in neonatal, infantile, late-onset, and transient forms with the neonatal form being the most severe and associated with high mortality and significant neurological impairment [3, 4]. Usually, NKH patients with the infantile form develop normally up to 6 months old, and then symptoms such as seizures, mental retardation, and chorea gradually appear. Symptom onset after 2 years of age is classified as late-onset NKH with a variety of potential problems, such as spastic paraparesis, optic atrophy, and neurologic degeneration. The transient form is rare and characterized by elevated levels of glycine in cerebrospinal fluid and plasma at birth, but glycine levels stay normal without any pharmacologic interventions from days to months [4–7]. The more severe the symptoms of NKH, the more difficult it becomes to manage seizures and airways. Diagnosis is confirmed through elevated glycine levels in plasma and cerebrospinal fluid, alongside genetic testing for mutations primarily in the GLDC and AMT genes.

Current treatment options, such as sodium benzoate and N-methyl-D-aspartate (NMDA) receptor antagonists like dextromethorphan and ketamine, offer limited efficacy and do not significantly alter disease progression. Additionally, there have been reports of delayed recovery of spontaneous breathing and prolonged emergence following the use of sevoflurane in patients with NKH [8–10].

In this case report, we discuss the anesthetic management of a patient with NKH using desflurane and nitrous oxide. This approach is proposed as an alternative to sevoflurane, given the patient’s history of delayed emergence from previous sevoflurane anesthesia. We aimed to explore the potential benefits of desflurane and nitrous oxide in improving anesthetic outcomes in NKH patients.

## Case presentation

Written informed consent was obtained from the patient’s guardian for the publication of this case report. At our institution, case reports are exempted from IRB approval.

A 6-year-old girl with NKH was scheduled for tracheostomy due to recurrent upper airway obstruction. The patient was born via cesarean section at 36 weeks gestation and required tracheal intubation soon after birth due to recurrent apnea. Myoclonus gradually appeared, leading to the diagnosis of NKH confirmed through genetic testing. At the age of 3 years, the patient underwent bilateral hip dissection for hip dislocation under general anesthesia with sevoflurane and continuous remifentanil infusion. During that surgery, she experienced hypoxemia post-extubation, and her emergence from anesthesia was delayed, taking 60 min from the end of surgery to her discharge from the operating room. By 4 years of age, the patient had begun continuous positive airway pressure therapy to manage severe obstructive sleep apnea.

At the preoperative examination for the tracheostomy, the patient’s height and weight were recorded as 82 cm and 17 kg, respectively. She experienced tonic convulsions lasting approximately 10 s several times a day, with accompanying SpO_2_ levels dropping to 60–70%. Blood tests and chest radiography revealed no significant abnormalities.

General anesthesia was induced with propofol (40 mg) and fentanyl (40 µg). Following induction, anesthesia was maintained with inhalation of 4% desflurane and 60% nitrous oxide. Rocuronium bromide (10 mg) was administered intravenously to facilitate tracheal intubation. After a few minutes, a train-of-four count remained at four, prompting the administration of an additional 5 mg of rocuronium. Despite limited mouth opening, tracheal intubation was successfully performed using a McGrath MAC video laryngoscope.

**Fig. 1:**
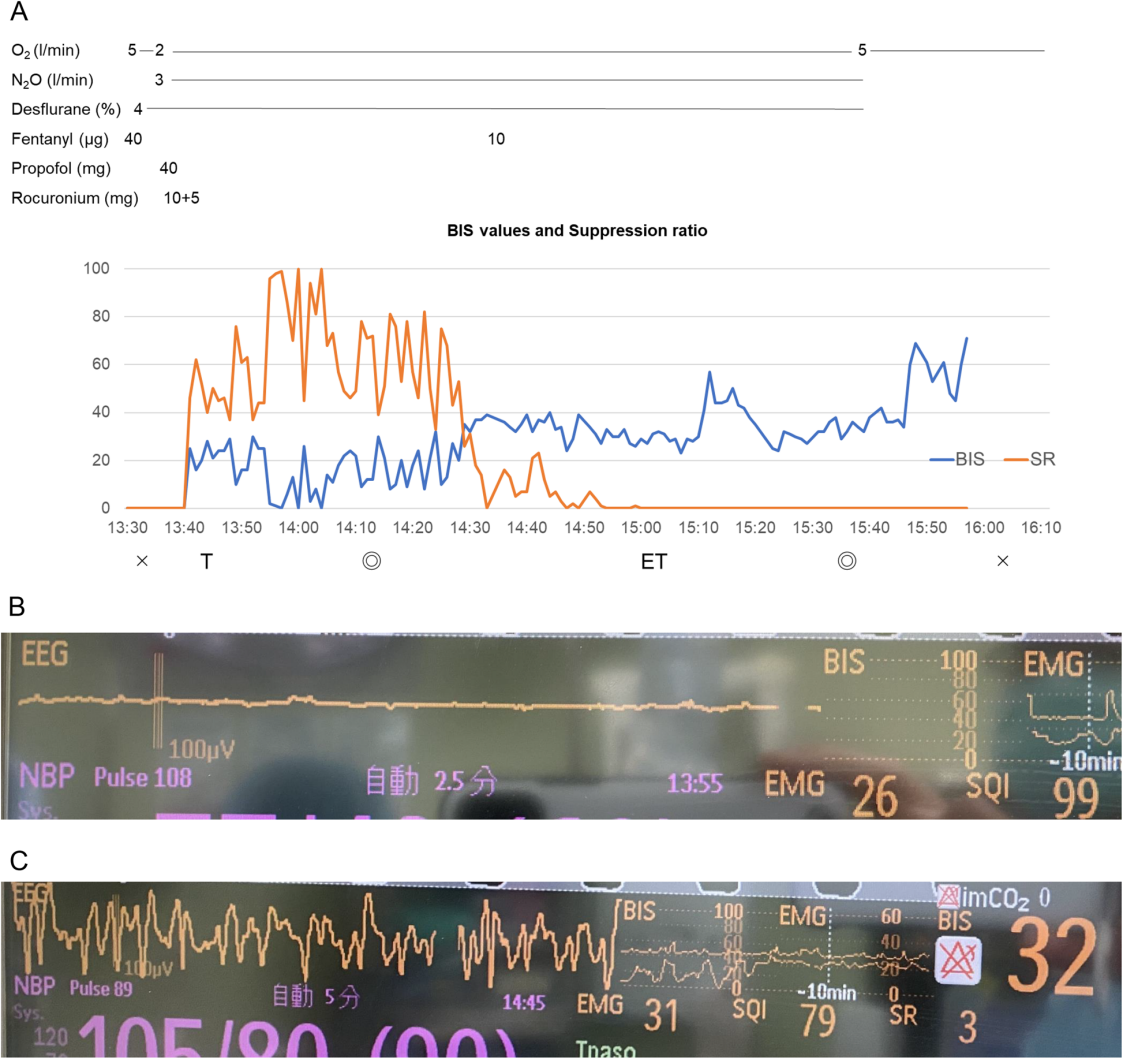
Anesthesia chart and bispectral index. **A** Anesthesia chart, bispectral index, and suppression ratio. **B**, **C** Electroencephalogram on bispectral index monitors around 30 min (**B**) and 90 min (**C**) after anesthesia induction

Throughout the procedure, hemodynamic parameters remained stable. However, the EEG showed significant suppression on the bispectral index (BIS) monitor (Fig. 1A, B), with a suppression ratio ranging from 40 to 60 for approximately 60 min. This suppression gradually decreased over time (Fig. 1C), and once it dissipated, the BIS value stabilized at approximately 40 until the desflurane was discontinued at the end of surgery. An additional 10 µg of fentanyl was administered intraoperatively. The concentrations of desflurane and nitrous oxide were kept constant throughout the anesthesia.

Following the cessation of desflurane and nitrous oxide, the patient opened her eyes 3 min later and spontaneous breathing resumed 19 min afterward. There were no instances of postoperative apnea or hypoxemia, and the frequency of seizures remained unchanged from preoperative levels. The patient was discharged from the hospital on the 14th postoperative day without any complications.

## Discussion

In this case, general anesthesia with desflurane and nitrous oxide facilitated early emergence in a patient with NKH, contrasting with her previous experience of delayed emergence following sevoflurane and remifentanil anesthesia. The patient’s almost flat EEG observed on the BIS monitor post-induction with propofol and fentanyl suggests a heightened sensitivity to anesthetic agents in NKH patients.

Previous reports have suggested that sevoflurane may cause delayed emergence in patients with NKH [9, 10], as seen in the patient’s history. Although desflurane has not been extensively studied in this patient population, the rapid recovery observed in this case supports its use as a potentially preferable anesthetic agent. Given that glycine is an inhibitory neurotransmitter in the spinal cord and brainstem, it can cause apnea and hiccups in NKH patients. However, NKH is thought to be neurotoxic owing to excessive activation of NMDA receptors produced by accumulated glycine which acts as a co-transmitter of glutamate [11]. However, we found no reports indicating a significant difference between the effects of sevoflurane and desflurane on glycine and NMDA receptors [12]. Therefore, we hypothesized that the delayed emergence produced by sevoflurane may be owing to its pharmacokinetic properties rather than to differences in the action of the drug itself.

The favorable pharmacological profile of desflurane, including its low blood-gas solubility coefficient, may also have contributed to the quicker emergence. The severe suppression on the BIS monitor observed in this case also suggests that patients with NKH may be more sensitive to sedative drugs, and we presume that the difference in the pharmacokinetic profile between sevoflurane and desflurane may have been expressed as a significant clinical difference.

Remifentanil, an ultra-short-acting opioid, is generally considered appropriate for NKH patients due to its rapid clearance [9]. However, the glycine present in clinical formulations of remifentanil posed a potential risk for this patient, given her condition’s underlying defect in glycine metabolism. This concern led us to avoid remifentanil, although there is no direct evidence to suggest that its use would be harmful in NKH patients.

Nitrous oxide, which has NMDA receptor antagonistic properties similar to ketamine [11, 13–15], was included in the anesthetic plan due to its potential to provide neuroprotective effects in NKH [16]. Although ketamine could theoretically alleviate symptoms related to NKH, its impact on the speed of emergence is unclear, and thus it was not used in this case.

After induction of general anesthesia with propofol and fentanyl, severe suppression of EEG signals was observed on the BIS monitor for approximately 60 min. Delayed emergence from sedation or anesthesia has been reported in patients with NKH [8–10]. Although the relationship between anesthetic sensitivity and preoperative EEG findings is unclear, abnormal EEG patterns, including burst suppression and hypsarrhythmia without the administration of sedatives, have been reported in neonatal and infantile cases of NKH [17]. The patient in the present case also exhibited burst suppression during the neonatal period and hypsarrhythmia at 5 months of age. In addition, although the concentrations of desflurane and nitrous oxide remained constant throughout the surgery, the suppression ratio decreased over time. The severe EEG suppression observed in this case may be due to the propofol used at the induction of anesthesia, indicating that NKH patients might be particularly sensitive to anesthetic agents. Ideally, we should compare the intraoperative EEG patterns with the preoperative ones. Unfortunately, preoperative EEG findings are unknown because an EEG recording had not been performed recently. Although we could confirm the EEG patterns before the induction of anesthesia, we regrettably attached the BIS sensor after the induction of general anesthesia. Therefore, it was difficult to conclude that the EEG patterns observed in the present case reflected the effect of general anesthetics including desflurane or propofol. However, the patients showed time-dependent recovery of the BIS value without any changes in desflurane concentration, suggesting that the effect of propofol could cause severe suppression of the EEG. However, in a previous report of a patient with NKH, the BIS value was 41 before anesthesia induction and varied from 32 to 55 under the administration of propofol, remifentanil, and nitrous oxide [9]. Although the symptoms, severity, and dosage of propofol in that case were similar to those in the present case, it is noteworthy that the intraoperative EEG findings differed. According to our study, we suggest that an anesthesiologist should monitor EEG before induction of general anesthesia and pay attention to the findings when a patient has a possibility of unpredictable sensitivity to general anesthetics.

In conclusion, this case suggests that desflurane and nitrous oxide, owing to their favorable pharmacological profiles, may be preferable anesthetic agents in patients with NKH, particularly those with a history of delayed emergence from sevoflurane anesthesia. The observed EEG suppression may reflect an increased sensitivity to anesthetics in patients with NKH.

## Data Availability

Not applicable.

## Abbreviations

BIS: Bispectral index
EEG: Electroencephalography
NKH: Non-ketotic hyperglycinemia
NMDA: N-methyl-D-aspartate
